# Health-related quality of life of among elders in rural China: the effect of widowhood

**DOI:** 10.1007/s11136-016-1338-y

**Published:** 2016-06-13

**Authors:** Jianfang Zhou, Norman Hearst

**Affiliations:** 1 Institute of Humanities and Social Sciences, Nanjing University of Posts and Telecommunications, Wenyuan Road 9, New Yadong District, Nanjing, 210023 China; 2 Departments of Family and Community Medicine and of Epidemiology and Biostatistics, University of California, San Francisco, CA 94143 USA

**Keywords:** Widowhood, QOL, Elders, Rural China

## Abstract

**Purpose:**

China has an enormous and rapidly growing population of widowed elders. Little is known about how losing a spouse affects elders’ health-related quality of life (QOL), especially in the rural areas where most Chinese elders live. This article analyzes QOL data collected in 2014 among rural Chinese elders to address this question.

**Methods:**

SF12 questionnaires and information about individual and household characteristics were collected from 3053 elders aged 60 and above in rural China. We compared the physical component summary (PCS) and mental component summary (MCS) scores between 1925 married elders and 1060 widowed elders in a bivariate model stratifying by gender and age group and in a general factorial ANOVA multivariate analysis that examined and controlled for other predictors of PCS and MCS scores including education, chronic disease, and family and household factors.

**Results:**

Widowed male and female elders’ physical health and mental health were in decline with age. Widowed men had lower PCS and MCS scores than married men. Widowed women also had lower PCS scores, but differences in MCS scores did not reach statistical significance. In multivariate analysis, widowhood was associated with lower PCS and MCS scores overall. Support from children was associated with better QOL and, based on interaction analysis, appeared to mitigate negative effects of widowhood.

**Conclusions:**

Widowed rural elders in China have lower physical and mental quality of life than their married counterparts. These elders rely on their children for care, and a supportive family is associated with better QOL.

## Background

Data from the Sixth Chinese National Population Census in 2010 show that there were 47.7 million widowed elders in China, accounting for 26.9 % of the total population over age 60, and that this rate was slightly higher (28.8 %) in rural areas. The number of widowed elders is expected to grow rapidly in China, reaching 118.4 million by 2050 [1]. The rapid increase in the number of elderly coupled with very low fertility rates in recent decades means that the number of elderly with few or no offspring to care for them will inevitably increase dramatically [2]. Therefore, the spouse will play a greater role in late life care. We conducted this study to examine the impact of widowhood on quality of life among elders in rural China.

Spouses play a protective role that might be expected to improve elders’ health-related quality of life (QOL) in ways ranging from daily care and accident prevention to spiritual consolation [3, 4]. QOL is a complex concept. The WHO QOL Group defined QOL as individuals’ perceptions of their position in life in the context of the culture and value systems in which they live and in relation to their goals, expectations, standards, and concerns [5]. Bowling and Gabriel postulate that QOL is a multi-dimensional collection of objective and subjective areas of life, the parts of which can affect each other as well as the sum [6]. An elder who loses a spouse may not only lose a certain degree of economic or social support. Widowhood may also bring loneliness and considerable difficulty in daily life. Assessing QOL of widowed elders and analyzing how losing a spouse affects elders’ QOL are thus important for designing effective health promotion strategies for widowed elders in China.

Research on QOL in China began fairly recently. A few studies of QOL among Chinese elders have been published in the past few years, though none has focused on the role of widowhood or been conducted in rural areas. These studies found that sex, age, and household economics were related to QOL [7–10]. Luo and Chen’s studies did not find marital status related to elders’ QOL [7, 8], but Feng and Tian’s studies showed that scores of both the physical and mental health components of quality of life of married elders were higher than unmarried elders (including single, divorced and widowed) [9, 10]. One study in urban China showed that more care from children was associated with better QOL [11]. Studies examining the relationship of living status and QOL among elders have given conflicting results. Cheng and Li’s study found that elders living with children had better overall QOL [12–14]. But Wei found that elders living only with their spouse scored better than those living with children [15].

Although no previous study has focused on Chinese widowed elders’ QOL, there are studies indicating increased standardized mortality among widowed elders, especially those newly widowed [16–20]. Numerous international studies have shown that married people have lower mortality than the unmarried [21–23]. Studies in England, Australia, and Norway [24–28] also found apparent effects of widowhood on physical and mental health and morale. But other studies [29, 30] found no health advantages for older women living with a spouse compared with those living alone. Some researchers suggest that reactions to bereavement are strongly influenced by culture and ethnicity [31, 32]. Different stages of bereavement also may have different effects on well-being, which in general tends to bounce back to its previous level following major adverse life events. How widowhood affects QOL of Chinese elders remains to be quantified, especially in rural areas. Better understanding this may help to explain subsequent morbidity and mortality and suggest ways to promote the health of these elders. This article analyzes QOL data collected in 2014 among rural Chinese elders to address these questions.

## Methods

### Study sample

The definition of rural elders in this study was a person age 60 and above who mainly lived in a rural area during the previous year. Since regional differences might influence QOL, this study sampled from three provinces, one each from the east (Jiangsu Province), the central (Henan province) and the western (Qinghai Province) regions of China. These provinces represent a range of social and economic rankings. One county was selected from each of the provinces by judgment sampling. The criteria were that in 2013 the county: (1) had more than 30,000 residents over age 60; (2) was mainly rural; and (3) had a social economy near the median level for the province. We selected two townships in each county by simple random sampling and obtained a list of 530 randomly selected families with elders using the local household registration information system in each township. We sampled 530 families to yield a total desired sample size of 3000 with a small margin of safety for non-respondents. We sampled two townships in each county because some rural townships have less than 1000 families with elders. For families with more than one elder, one was randomly selected. We successfully interviewed a total of 3053 elders. For the 4.0 % of the sample not interviewed (the rate was similar in married and widowed elders), the main reason was not being at home during the approximately one-week period of interviews. Among the participants, there were 1925 married elders, 1060 widowed elders, and 68 single elders. The 68 single elders who were not widowed were excluded from the study.

### Data collection method

This study was approved by the Nanjing University of Posts and Telecommunicates Ethics Committee. All participants gave informed consent prior to inclusion in the study. After conducting a pretest in one of the sample counties in January 2014, formal investigation took place from March to May. Local trained investigators interviewed elders (or their caregivers for the 8.3 % of elders who could not answer questions by themselves due to their health status) at their home. In each county, we selected 30 interviewers from local community workers. The main criteria were: (1) age 30–50; (2) junior high school education or higher; (3) availability to work full time during the survey week; and (4) good patience, communication skills, and responsibility.

### Variables

#### Individual characteristics

Based on previous studies [7–9, 33–40], this study collected data on respondents’ date of birth (this was converted into age in years and analyzed in age strata and as a continuous variable); sex; highest level of education (illiterate, elementary school, junior high school or higher); whether suffering from chronic diseases (yes vs. no); and current marital status (married vs. widowed). For those widowed, we asked the year in which the spouse had died; we did not ask the exact date because our pre-test indicated that widows often could not remember this.

#### Household factors

We constructed questions to assess household factors based on variables associated with QOL in previous research [34–39]. Participants were asked: whether they lived with at least one of their children (yes vs. no); frequency of children asking about their health (always, often, seldom, or never asked); whether children were one of their main source of income (yes vs. no); and whether children were one of their main caregivers when they were ill (yes vs. no).

QOL This study used SF-12 (V2 in Chinese) to measure the health-related QOL of elders. The scale was developed by the Health Research Institute of the New England Medical Center in Boston, USA. It comprehensively summarizes the content of physical, psychological, and social aspects of health [40]. The scale is composed of 12 items divided into eight dimensions: physical functioning (PF), role physical (RP), bodily pain (BP), general health (GH), vitality (VT), social functioning (SF), role emotional (RE), and mental health (MH). Scoring was based on the scale users’ manual [40]. The range of each dimension score was from 0 to 100 with higher scores meaning higher QOL. From these scores, the physical component summary (PCS) and mental component summary (MCS) scores were also calculated based on the manual. A score higher than 50 represents above the original norm (USA) and higher scores mean higher QOL. Lam tested SF12 V2 (Chinese version) in the general Chinese population and confirmed it had good reliability and validity [41]. Xiao tested it on Chinese elders who lived in pension institutions or at home and also confirmed that it had good reliability and validity. [42]

### Analysis

We used Epidata 3.2a to record responses and SPSS 17.0 for analysis. Interviewers and investigators double-checked all questionnaires in the field, so no questionnaire items were missing for more than 1 % of respondents. In the few cases of missing data, we substituted the mode value (for qualitative variables) or mean value (for quantitative variables) in accordance with the SF12 users’ instructions and for consistency with other studies. We compared the differences of the personal and household factors and PCS and MCS scores between widowed and married elders using the Chi-square test and one-way ANOVA. General factorial ANOVA analysis was then applied to examine the association of widowhood with PCS and MCS scores in a multivariate model to control for the effect of potential confounding variables. A second ANOVA model added interaction terms for widowhood with other significant predictors of QOL including age, sex, education, chronic disease, and family factors. The results of effects (measures of association) are shown as mean square, *F* and *P* value. Two-tailed *P* < 0.05 is considered significant.

## Results

### Individual and household characteristics

As shown in Table 1, most of the widowed elderly participants were illiterate women with chronic diseases. Their average age was 73.5 years. Most of them lived with their children. Nearly 40 % of them relied on their children as a main source of living expenses. Most reported that their children often asked about their health and cared for them when they were ill. Compared to married elders, widowed elders’ personal characteristics showed significant differences: more were women, they were older, and more of them had chronic diseases. Judging from the individual characteristics, widowed elders had less personal health resources than the married elders. On the other hand, children provided more financial support and care to widowed elders than married elders. More of the widowed reported that their children were a main source of financial support, lived with them, and were a main caregiver when they were ill.

**Table 1:** Comparison of personal and family characteristics between widowed and married elders

Variable	Married elders (*n* = 1925)	Widowed elders (*n* = 1060)	*P* value
Sex	Male 57.2 %, Female 42.8 %	Male 29.7 %, Female 70.3 %	<0.001
Age	Average age 67.51, Standard deviation 6.01	Average age 73.51, Standard deviation 8.03	<0.001
Education	Illiterate 50.8 %; Elementary school 34.8 %; Junior high school or higher 14.4 %	Illiterate 71.0 %; Elementary school 22.2 %; Junior high school or Higher 6.8 %	<0.001
Having chronic diseases	Yes 82.4 %; No 17.6 %	Yes 85.8 %; No 14.2 %	<0.001
Living with children	Yes 37.7 %; No 62.3 %	Yes 52.8 %; No 47.2 %	<0.001
Children as a main source of financial support	Yes 21.4 %; No 78.6 %	Yes 41.9 %; No 58.1 %	<0.001
Frequency of children asking about their health	Frequently 70.6 %; Once in a while 24.2 %; Almost never 5.1 %	Frequently 71.0 %; Once in a while 23.9 %, Almost never 5.4 %	0.577
Children as a main caregiver when ill	Yes 70.7 %; No 29.3 %	Yes 93.2 %; No 6.8 %	<0.001

For married elders, 89.7 % also reported that their partners were a main caregiver for them when they were ill. The percentage of children who were a main caregiver when ill was somewhat higher for female elders than male elders (74.4 % vs 67.9 %; *P* = 0.010 by Chi-square analysis; respondents could select more than one main caregiver when ill.)

Our sample included relatively few elders who were recently widowed, especially among the older age groups. Overall, only 5.7 % had been widowed for less than a year and 71.9 % had been widowed for over 5 years. The percentage widowed over 5 years reached a maximum of 81.0 % among widowers age 80 or higher. The small numbers recently widowed in many age/sex strata and the collinearity of length of widowhood with age precluded separate analysis of the effect of time since death of a spouse on QOL.

### QOL comparison between widowed elders and married elders

Since age and gender are strong predictors of QOL and because previous studies have suggested widowhood may have different impact on QOL by sex and age [16–24, 28], we first compared QOL of married and widowed elders stratifying by gender and age group.

#### Males

**Fig. 1:**
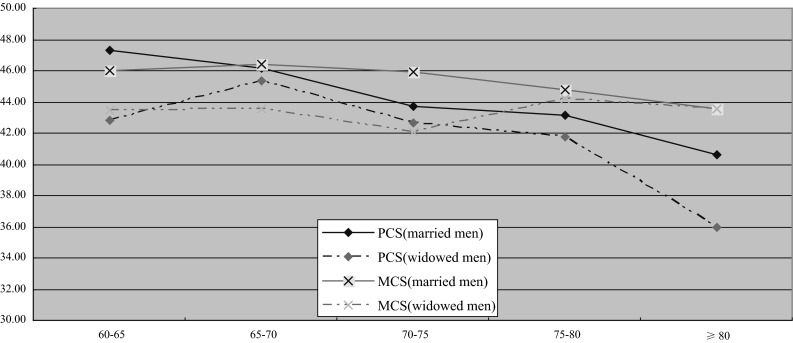
QOL comparison between widowed and married men by 5-year age group

Widowed male elders’ physical health and mental health were in decline with age, but the decrease in physical health was more apparent (Fig. 1). One-way ANOVA analysis showed that compared with the male married elders, widowed men had lower PCS and MCS scores in general after controlling for age group (*P* < 0.01). When looking within specific age groups, we found that the PCS scores of widowed elders were significantly lower in the 60–65 and ≥80 years age groups. The MCS scores of widowed elders were significantly lower in 60–65, 65–70 and 70–75 years age groups.

#### Females

**Fig. 2:**
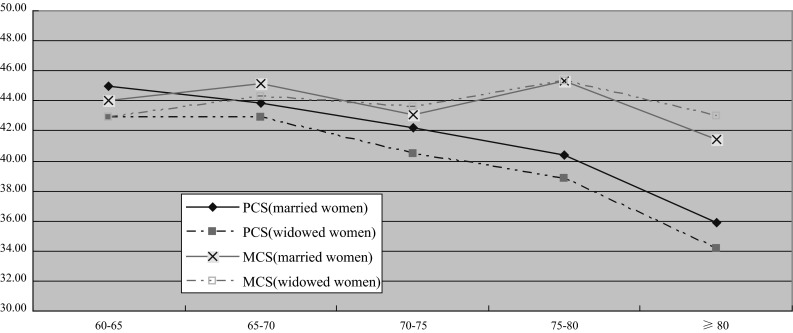
QOL comparisons between widowed and married women by 5-year age groups

QOL of both widowed and married female elders also declined significantly with age. Their level of PCS dropped faster than MCS especially after age 70 (Fig. 2). In contrast to male elders, differences in MCS scores between widowed and married women did not reach statistical significance, either overall or within any age group. PCS was lower overall (*P* < 0.01) and in the 60–65 age group.

### General factorial ANOVA analysis of the association of widowhood with PCS and MCS

The comparison above shows that widowed elders’ physical and mental health status tend to be poorer than married elders of the same sex and age groups, except for MCS among women. We used general factorial ANOVA analysis to examine the extent to which these differences might be due to confounding by other individual and family factors (Table 2).

**Table 2:** General factorial ANOVA analysis of predictors of PCS and MCS (without interaction terms)

	PCS			MCS		
	Mean square	*F*	*P* value	Mean square	*F*	*P* value
Sex	746.68	8.54	0.004	8.27	0.12	0.729
Age	3607.45	41.25	<0.001	205.18	2.97	0.018
Married versus widowed	906.38	10.36	0.001	279.48	4.01	0.037
Education	597.24	6.83	0.001	873.97	12.65	<0.001
Having chronic diseases	22,225.99	254.14	<0.001	5058.64	73.24	<0.001
Living with children	51.76	0.59	0.442	4.32	0.06	0.803
Children as a main source of financial support	638.57	7.30	0.007	1043.41	15.11	<0.001
Frequency of children asking about their health	491.82	5.62	0.004	2350.38	34.03	<0.001
Children as a main caregiver when ill	136.90	1.57	0.211	762.06	11.03	0.001
Overall model significance	Mean square = 4344.92; Sig. < 0.001			Mean square = 1302.91; Sig. < 0.001		

Personal characteristics such as sex, age, marital status, education, and whether having chronic diseases were all significantly related to PCS. Elders who were female, older, widowed, illiterate, and with chronic diseases all scored lower. If their children asked more often about their health and were not a main source of financial support, they scored higher.

For MCS, elders who were older, widowed, illiterate, and with chronic diseases scored lower. If their children asked about their health more often, were a main caregiver, or were a main source of financial support, they scored higher.

Table 3 presents a second multivariate model that includes interaction terms between marital status and other predictors of QOL including age, sex, education, chronic disease, and family factors. In this model, marital status was not directly associated with PCS or MCS, but several interactions between marital status and other predictors of QOL were significant. For PCS, those who were uneducated, who did not live with their children, and whose children seldom asked about their health suffered more from widowhood. For MCS, those who were male, who did not live with their children, and whose children were a main source of financial support suffered more from widowhood.

**Table 3:** General factorial ANOVA analysis of predictors of PCS and MCS (including interaction terms)

	PCS			MCS		
	Mean square	*F*	*P* value	Mean square	*F*	*P* value
Sex	231.73	2.69	0.101	0.42	0.01	0.937
Age	5202.35	60.44	0.000	253.96	3.71	0.025
Married versus widowed	11.17	0.13	0.719	6.80	0.10	0.753
Education	942.42	10.95	0.000	579.68	8.47	0.000
Having chronic diseases	21,098.13	245.11	0.000	4110.25	60.03	0.000
Living with children	220.73	2.56	0.109	99.07	1.45	0.229
Children as a main source of financial support	598.12	6.95	0.008	1308.36	19.11	0.000
Frequency of children asking about their health	325.94	3.79	0.023	1787.60	26.11	0.000
Children as a main care giver when ill	122.88	1.43	0.232	39.49	0.58	0.448
Sex × marriage	259.71	3.02	0.082	355.51	5.19	0.023
Age × marriage	73.74	0.86	0.425	84.72	1.24	0.290
Education × marriage	332.82	3.87	0.021	11.45	0.17	0.846
Chronic disease × marriage	202.42	2.35	0.125	16.12	0.24	0.628
Living with children × marriage	1389.14	16.14	0.000	429.85	6.28	0.012
Marriage × Children as a main source of financial support	120.64	1.40	0.237	1239.00	18.09	0.000
Frequency of children caring about health × marriage	324.80	3.77	0.023	160.79	2.35	0.096
Children as main care givers when illness × marriage	0.02	0.00	0.988	245.50	3.59	0.058
Overall model significance	Mean square = 2849.12; Sig. < 0.001			Mean square = 914.09; Sig. < 0.001		

## Discussion

### Overall QOL scores

Mean scores for both MCS and PCS in both sexes and all age groups were below the score of 50 that represents the original norm of the scale. The SF12 scale has been shown to have good reliability and validity in China, but it has not yet been normalized for Chinese populations, let alone specifically for this age group. “Low” mean scores might be due to many factors including age, nuances in wording of translated questions, and/or cultural factors in how respondents answer questions when interviewed. For example, in some cultures elders might gain sympathy by complaining about their health, while in others they might gain respect by being more stoic. Chinese aging populations generally tend to score below international norms on QOL scales [7–9], but this may or may not mean that their level of health-related quality of life is truly lower. Perhaps all that can be said is that there is no indication from these results that overall health-related QOL in these elders is especially high.

### Widowhood and QOL

As hypothesized, QOL was lower in widowed elders than in married elders in most comparisons. This difference was statistically significant in the initial analysis stratified by age and sex for PCS in both men and women and for MCS in men as well as being significant for both PCS and MCS in the combined multivariate model. When looking within age strata, these differences tended to be greater for younger elderly widows than for the older elderly, with the exception of PCS for men, which also showed a significant difference in the oldest age group.

MCS was not significantly different overall between widowed and married women. Inspection of Fig. 2 shows a nonsignificant trend toward worse MCS for widowed women for ages 60–69, which disappears by age 70. We suspect the overall nonsignificant result may have been due to our study including a substantial number of older widowed women who had been widowed for many years and who had time to adjust psychologically to widowhood in the context of a supportive family environment. This would be consistent with other research suggesting that some of the negative effects of widowhood may decline with time [26, 43]. MCS in men showed a similar pattern, but differences in age groups under 75 were enough to produce a significant difference for all ages combined. In our study, widowed women tended to be older than widowed men, had probably been widowed for longer, and were more likely to be cared for by their children, perhaps attenuating the impact of widowhood on MCS more in women than in men.

These results are generally consistent with other studies on the effect of widowhood in China and internationally. Chinese studies of widowhood and mortality indicate that men suffer more from widowhood [11–14], as has also been found in England, although studies in North America and Europe did not find differences by gender [16]. This may be due to cultural differences, as previous studies have suggested [31, 32]. In China, women rely on their adult children more than men, who are supposed to be independent, especially economically.

### Other predictors of QOL

Although this was not the main focus of this study, our multivariate analysis identified several other predictors of health-related QOL among these rural Chinese elders, including age, sex, education, and having chronic diseases. These results are consistent with previous international QOL studies among elders [34–38]. Similar to findings in the Chinese middle-aged population [39], family-related characteristics, such as frequency of children’s asking about health and whether children are a main source of financial support and care when ill, were also significant predictors of elders’ QOL. These results are mostly consistent with a previous study in urban China [11]. One exception is that PCS was higher for elders when children were not a main source of financial support in our study. This may be because elders in better physical health were able to support themselves and did not need financial support from their children.

According to our multivariate analysis, living with children was not by itself a significant predictor of QOL. But it interacted significantly with marital status such that widowed elders had poorer PCS and MCS when they did not live with their children. This is also consistent with previous studies of Chinese elders [12–14]. In general, our interaction analysis suggests that in our sample of elders, who had mostly been widowed for many years, the negative effects of widowhood on quality of life can be mostly mitigated by having a supportive family and to a lesser degree by education.

### Limitations of this study

This study has many limitations. The cross-sectional design can detect associations but cannot identify cause and effect. The measures we used for QOL, while in line with international standards, have not been normalized for elderly Chinese populations, and responses may be biased for the 8.3 % of elders for whom questions were answered by caregivers. Our measures of the family factors that appear to be of great importance are relatively crude. Although our sample was designed to give a broad spectrum of rural environments in China, it does not provide a basis for weighting results to make them representative of rural China overall. Although our sample was fairly large, numbers in age–sex strata were smaller, so that apparent differences by subgroup should be viewed with caution.

## Conclusions

Our results raise concerns for the enormous and rapidly growing population of widowed rural Chinese elders, who have lower physical and mental quality of life than their married counterparts. These elders rely on their children for care, and a supportive family does indeed seem to mitigate negative effects of widowhood on both PCS and MCS. But the rapid increase in the elderly population in rural China coupled with drastically lower fertility rates in recent decades raises questions about whether this model is sustainable. Increasingly, an adult couple may be the only descendants of up to four elders. If this couple migrates to a big city for work or is otherwise unable or unwilling to care for their parents, these elders may have no descendants to care for them at all.

A combination of approaches may be needed to address this situation in the future. Families should be provided with encouragement, resources, and support to help them care for their elders. This might include help from government with the financial burden, visiting nurses to help deal with medical issues and daytime or respite care as is provided for elders in some other countries. In addition, a strengthened social safety net must be prepared to care for elders who are no longer able to care for themselves and who do not have family who are willing and able to care for them. This is especially true for widowed elders who may otherwise be left to face the world alone in declining physical and emotional health.
